# Identification of Genetic Loci for Sugarcane Leaf Angle at Different Developmental Stages by Genome-Wide Association Study

**DOI:** 10.3389/fpls.2022.841693

**Published:** 2022-05-27

**Authors:** Xinglong Chen, Zhenghui Huang, Danwen Fu, Junteng Fang, Xiangbo Zhang, Xiaomin Feng, Jinfang Xie, Bin Wu, Yiji Luo, Mingfeng Zhu, Yongwen Qi

**Affiliations:** ^1^Institute of Nanfan & Seed Industry, Guangdong Academy of Sciences, Guangzhou, China; ^2^College of Agriculture and Biology, Zhongkai University of Agriculture and Engineering, Guangzhou, China

**Keywords:** sugarcane, leaf angle, GWAS, InDel marker, candidate genes

## Abstract

Sugarcane (*Saccharum* spp.) is an efficient crop mainly used for sugar and bioethanol production. High yield and high sucrose of sugarcane are always the fundamental demands in sugarcane growth worldwide. Leaf angle and size of sugarcane can be attributed to planting density, which was associated with yield. In this study, we performed genome-wide association studies (GWAS) with a panel of 216 sugarcane core parents and their derived lines (natural population) to determine the genetic basis of leaf angle and key candidate genes with +2, +3, and +4 leaf at the seedling, elongation, and mature stages. A total of 288 significantly associated loci of sugarcane leaf angle at different developmental stages (eight phenotypes) were identified by GWAS with 4,027,298 high-quality SNP markers. Among them, one key locus and 11 loci were identified in all three stages and two stages, respectively. An InDel marker (SNP Ss6A_102766953) linked to narrow leaf angle was obtained. Overall, 4,089 genes were located in the confidence interval of significant loci, among which 3,892 genes were functionally annotated. Finally, 13 core parents and their derivatives tagged with SNPs were selected for marker-assisted selection (MAS). These candidate genes are mainly related to MYB transcription factors, auxin response factors, serine/threonine protein kinases, etc. They are directly or indirectly associated with leaf angle in sugarcane. This research provided a large number of novel genetic resources for the improvement of leaf angles and simultaneously to high yield and high bioethanol production.

## Introduction

Sugarcane (*Saccharum* spp.) is the major raw material for the global sucrose supply and the preeminent energy crop for bioethanol production (Dahlquist, 2013). High biomass yield and high sucrose content are fundamental demands for sugarcane production. Plant architecture is one of the most important characteristics determining the yield of plant. Donald first proposed the concept of ideal plant architecture in 1968 (Donald, 1968). It is necessary to find the plant architecture that confers the least competition among individuals in the field, which can maximize the utilization of light energy and increase the plant yield. Leaf angle is an important component of sugarcane plant architecture as it can determine how light is acquired and the spatial distribution of the leaves. When the leaf angle is narrow, the blade is vertically oriented, and the plant type is compact, while when the leaf angle is large, the blade is horizontally oriented, and the plant type is spreading leaf. Thus, leaf angle can be optimized to reasonably improve the planting density and photosynthetic efficiency of sugarcane, which is an effective method to increase the crop yield. This has been widely used in rice, maize, wheat, and other crops (Isidro et al., 2012; Li et al., 2015; Zhang et al., 2015).

The regulation mechanism of leaf angle is a complex process. At present, many studies have shown that leaf angle regulation is mostly related to plant hormone synthesis and signal transduction (Tong et al., 2012; Sun et al., 2015; Miao et al., 2016). The inhibition of brassinosteroid (BR) synthesis will lead to the decrease in leaf angle in rice (Yamamuro et al., 2000), while the overexpression of key synthetic genes will lead to the increase in leaf angle (Tanabe, 2005). In terms of BR signal transduction, leaf angle mutants d2-2 and d61-1 were inhibited by U-type cyclin (CYCU4; 1), which enhanced the proliferation of a group of special cells on the far axis of the occipital region, resulting in the upright leaves (Sun et al., 2015). On the contrary, the overexpression and activation of key genes *DLT, GSK2, BU1*, and *ILI1* of the signal pathway will lead to the enlargement of leaf angle and oblique downward extension of leaves (Tanaka et al., 2009; Tong et al., 2009; Wan et al., 2009; Zhang et al., 2011). In addition to BR, auxin (IAA), gibberellins (GA), and ethylene are also involved in the regulation mechanism of rice leaf angle (Qiao et al., 2009; Hirano et al., 2010; Zhao et al., 2010; Miao et al., 2016), and some are not related to hormones, for example, *OSDCLl3* regulates the enlargement of rice leaf angle by producing a segment of 24-ntSiRNAs (Wei et al., 2014). Other similar genes such as *OsWRKY11, OsLG1*, and *OsARF19* can participate in the regulation mechanism of leaf angle (Wang et al., 2005; Lee et al., 2007; Zhang et al., 2015). Moreover, microRNA has also mediated of leaf angle. Overexpression of miR393 alters an auxin signaling pathway by inhibiting the transcription of target genes *OsAFB* and *OsTIR1*, due to the increase in flag leaf angle in rice (Bian et al., 2012). Overexpression of miR1848 and RNAi *OsCYP51G3* led to the decrease in leaf angle in rice (Xia et al., 2015).

The mechanism of leaf angle regulating leaf angle in sugarcane has been rarely reported, mainly due to the complex genetic background of sugarcane. However, with the rapid development of sequencing technology and the high-density SNP markers, genome-wide association study (GWAS) has become the most powerful method to explore the quantitative characteristics of sugarcane. Furthermore, GWAS has been instrumental in important breakthroughs in sugarcane yield-related traits, sugar content, and fiber fraction (Banerjee et al., 2015; Gouy et al., 2015; Fickett et al., 2019; Yang et al., 2019). For the complicated trait of leaf angle, GWAS should be suitable to identify loci that contribute to this trait. The objective of this study was (i) to figure out the distribution of leaf angle in this panel of 216 sugarcane core parents and their derived lines (natural population) at the seedling, elongation, and mature stages; (ii) to identify the loci significantly associated with leave angle by GWAS; and (iii) to identify possible candidate genes by annotating these loci, which could provide genetic resources useful for the improvement in leaf angle and MAS in sugarcane.

## Materials and Methods

### Plant Materials and Growth

In this study, a panel of 216 core parents and their derivatives were selected to construct a sugarcane natural population (Supplementary Table 1). They were derived from many sugarcane planting countries, namely, China (150), USA (32), Australia (10), India (4), Cuba (7), Brazil (2), France (1), Philippines (4), Mauritius (2), South Africa (2), Thailand (1), and Indonesia (1). Among the 216 materials, 204 materials have been used as parents in breeding and 12 are newly bred materials used as parents. According to our previous research, 163 varieties were bred from 21 parents, including CP49-50, F134, Co419, CP72-1210, CZ2, NCo310, F108, HN56-12, YC71-374, YN73-204, and CP28-11, accounting for 87.63% of all the varieties worldwide. The varieties bred in recent 10 years are mainly bred by the offspring of these 21 varieties. Among the 21 core parents, CP49-50, Co419, and NCo310 are traditional and elite varieties used commercially all over the world (Zhang et al., 2009), and most of them were selected as breeding parents in China. This group represents an important genetic background in China.

The natural population was planted in Wengyuan base of the Institute of Nanfan & Seed Industry, Guangdong Academy of Sciences (Guangzhou Sugarcane Research Institute), in 2019. The base is located at 24° 17′ 00″ N and 113° 56′ 25″ E with an altitude of 120 m. The experimental design consisted of a completely randomized group with two repeats. Practices were used to ensure that seedling emergence was regular and the spacing of each seedling was uniform. Before planting, seedlings were disinfected, cut into single buds, and transplanted to the field after the seedlings grew to 20 cm at a row spacing of 1.1 m and a plant spacing of 25 cm. There were three rows of repeat planting and 16 plants in each row. Only 10 plants in the middle of the plots were investigated. The field management was carried out according to conventional field management, with normal fertilization, irrigation, and control of diseases, pests, and weeds.

### Phenotype and Statistical Analysis

Due to the leaves at the seedling stage being few in number and the +1 leaf being too close to the heart leaf for facile measurement, the leaf angle of the +2 leaf and +3 leaf of each accession was measured at the seedling stage (roughly 2 months old) of the natural population. The leaf angle of the +2 leaf, +3 leaf, and +4 leaf of each accession was measured at the elongation and mature stages, respectively. The phenotypic data were analyzed by Excel 2010.

### The Whole-Genome Resequencing and Genotyping

The genomic DNA of this natural population was extracted according to the method described by Aljanabi et al. (1999). Resequencing was performed by the Beijing Nuohe Zhiyuan Bioinformatics Technology Co., Ltd., with a sequencing depth of 5× using an Illumina Hiseq 2500. The raw reads filtered out those corresponding to adapter sequences, those with more than a 10% N content, and those <10 bases and selected those with more than a 50% quality value. All sequencing data were aligned to the *Saccharum spontaneum* reference genome (Zhang et al., 2018) by BWA software (fast and accurate short read alignment with the Burrows–Wheeler transform; Giannoulatou et al., 2014), and PCR duplicates reads were further removed by SAMtools (v1.3.167, the Sequence Alignment/Map format and SAMtools; Zhao et al., 2020). SNPs were identified among 216 samples by the HaplotypeCaller module of GATK (v3.868) in GVCF mode (The Genome Analysis Toolkit: a MapReduce framework for analyzing next-generation DNA sequencing data; Bernhardsson et al., 2020). Then, a joint call was performed using GATK GenotypeGVCFs for all 216 samples. SNPs were filtered using the GATK VariantFiltration function with the parameter “QD <2.0| | FS > 60.0| | MQRankSum < −12.5| | ReadPosRankSum < −8.0| | SOR > 3.0| | MQ <40.0| | DP > 30| | DP <3.” SNPs with a minimum allele frequency (MAF) ≥5% and a missing rate ≤ 50% were kept for downstream analysis.

### Linkage Disequilibrium Analysis

Linkage disequilibrium (LD) *r*^2^ was calculated using SNPs with MAF > 0.05 and a missing rate <0.5 by PLINK (v.1.90b3.42, PLINK: a toolset for whole-genome association and population-based linkage analyses) with the following parameters: –ld-window-*r*^2^ 0 –ld-window 99999 –ld-window-kb 500 (Sadowski et al., 2019). The genome-wide average *r*^2^ between two SNPs within 500-kb windows was calculated, and the distance of LD decay was represented as the physical distance over which the *r*^2^ drops to 0.1.

### Genome-Wide Association Study

SNPs were imputed by the Beagle software with default parameters (a one-penny imputed genome from the next-generation reference panel; Khvorykh and Khrunin, 2020). Kinship was analyzed by an emmax-kin module in emmax software with the parameters of -v -h -s -d 10 (variance component model to account for sample structure in genome-wide association studies; Li et al., 2019). We used the admixture software (http://software.genetics.ucla.edu/admixture/, v1.3.0) to perform population structure analysis. GWAS was carried out using the Emmax software in a linear mixed model with kinship matrix and population structure (variance component model to account for sample structure in genome-wide association studies; Pino Del Carpio et al., 2018). The number of independent SNPs was calculated, which was used to determine the genome-wide significance cutoff of GWAS. Finally, the significance cutoff of GWAS was -log(P) ≥ 6.

### Genomic DNA Extraction and PCR Amplification

Genomic DNA was extracted using the Plant genomic DNA kit (Tiangen, Beijing, China) according to the manufacturer's protocols. DNA sample was examined on 1.0% agarose gels and quantified by spectrophotometer.

**Figure 1:**
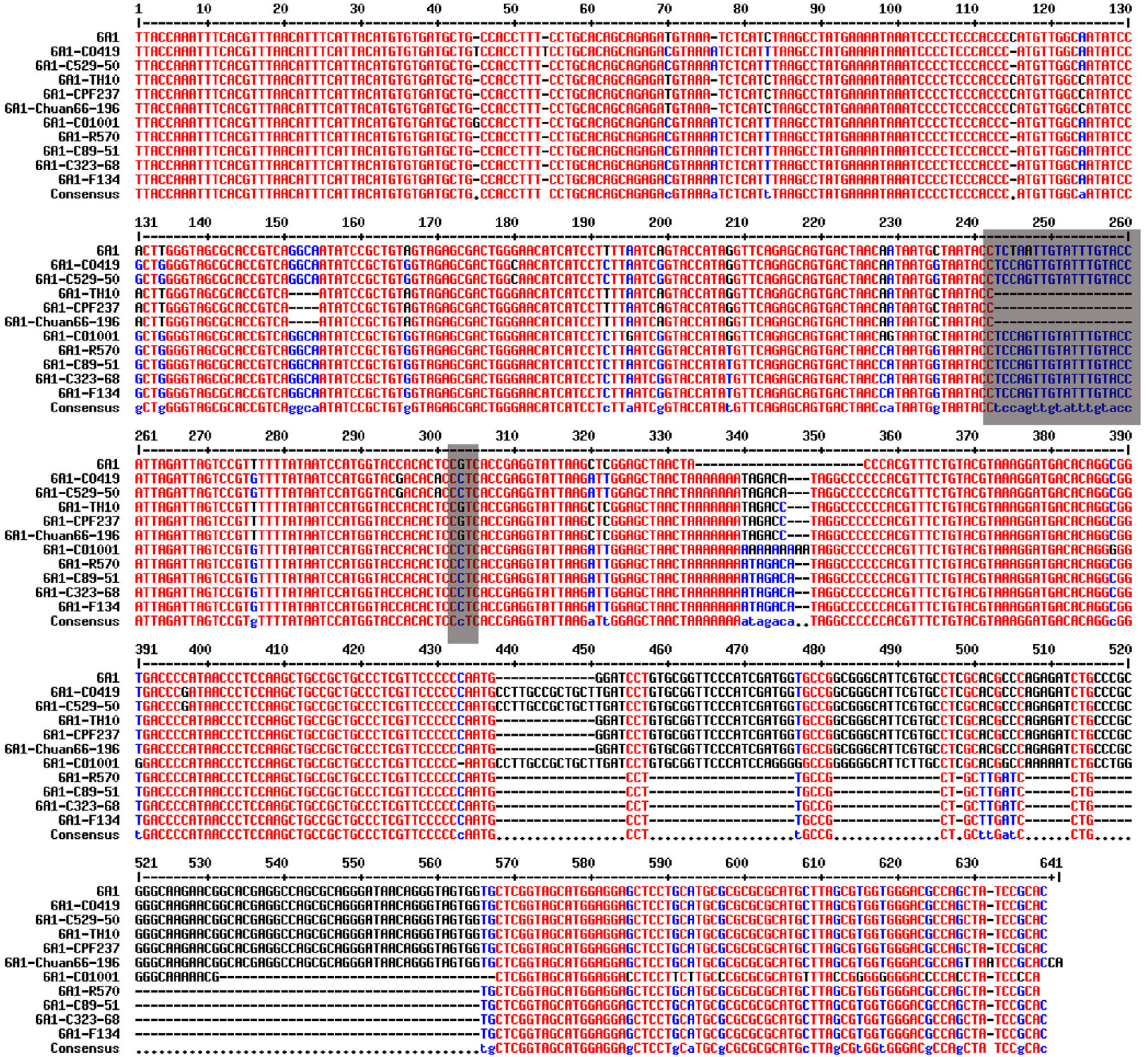
Blast results of amplification sequences of SNP SS6A_102766953-G-C from 10 sugarcane core parents and their derived lines.

The sugarcane cv. R570, C89-51, C323-68, CO419, C529-50, CO1001, TH10, CPF237, F134, and Chuan66-196 genomic DNA were selected to run the PCR amplification. Primers were designed 300 bp before and after the SNPs according to the sequences. PCR products of each reaction were run on 1% agarose gels, and a single fragment was recovered from gels and purified using a DNA purification kit (Magen, Beijing, China). The fragment was ligated into the plasmid, transformed into Escherichia coli DH5α competent cells (Weidi, Shanghai, China), and then sequenced (Sangon, Shanghai, China). The primers used for PCR are listed in Supplementary Table 2. The 12 SNP markers and the sequences used for PCR amplification are listed in Supplementary Material. The blast results of amplification sequences of SNP SS6A_102766953-G-C from 10 sugarcane varieties are shown in Figure 1.

### Candidate Gene for Associated SNPs

We started by merging significant SNPs based on an *r*^2^ measure of LD ≥ 0.3. We then defined the confidence intervals as the 250-kb flanking region around each LD block. Genes located within the confidence interval were classified as candidate genes. InterProScan software (v5.39-77.0) with default parameters was applied to the annotated genes in *S. spontaneum* with the protein sequences as input files.

## Results

### Phenotypic Analysis of Leaf Angle in Sugarcane Natural Population

**Figure 2:**
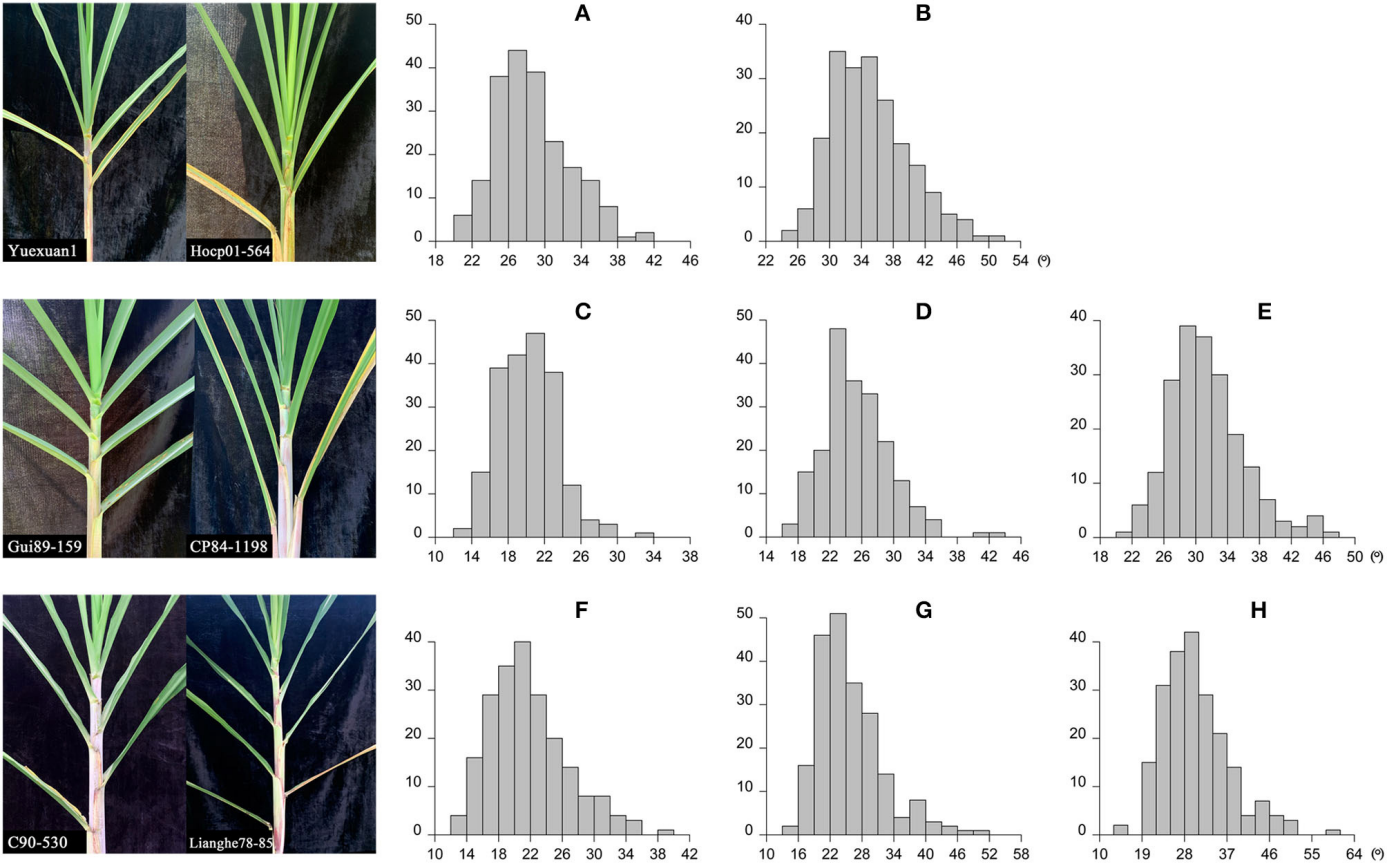
Leaf angle and the frequency distribution in sugarcane different growth stages. **(A,B)** Indicate the +2 and +3 leaf angle in the seedling stage, respectively (206 accessions); **(C–E)** indicate the +2, +3, and +4 leaf angle in the elongation stage, respectively (203 accessions); **(F–H)** indicate the +2, +3, and +4 leaf angle in the mature stage, respectively (211 accessions).

**Figure 3:**
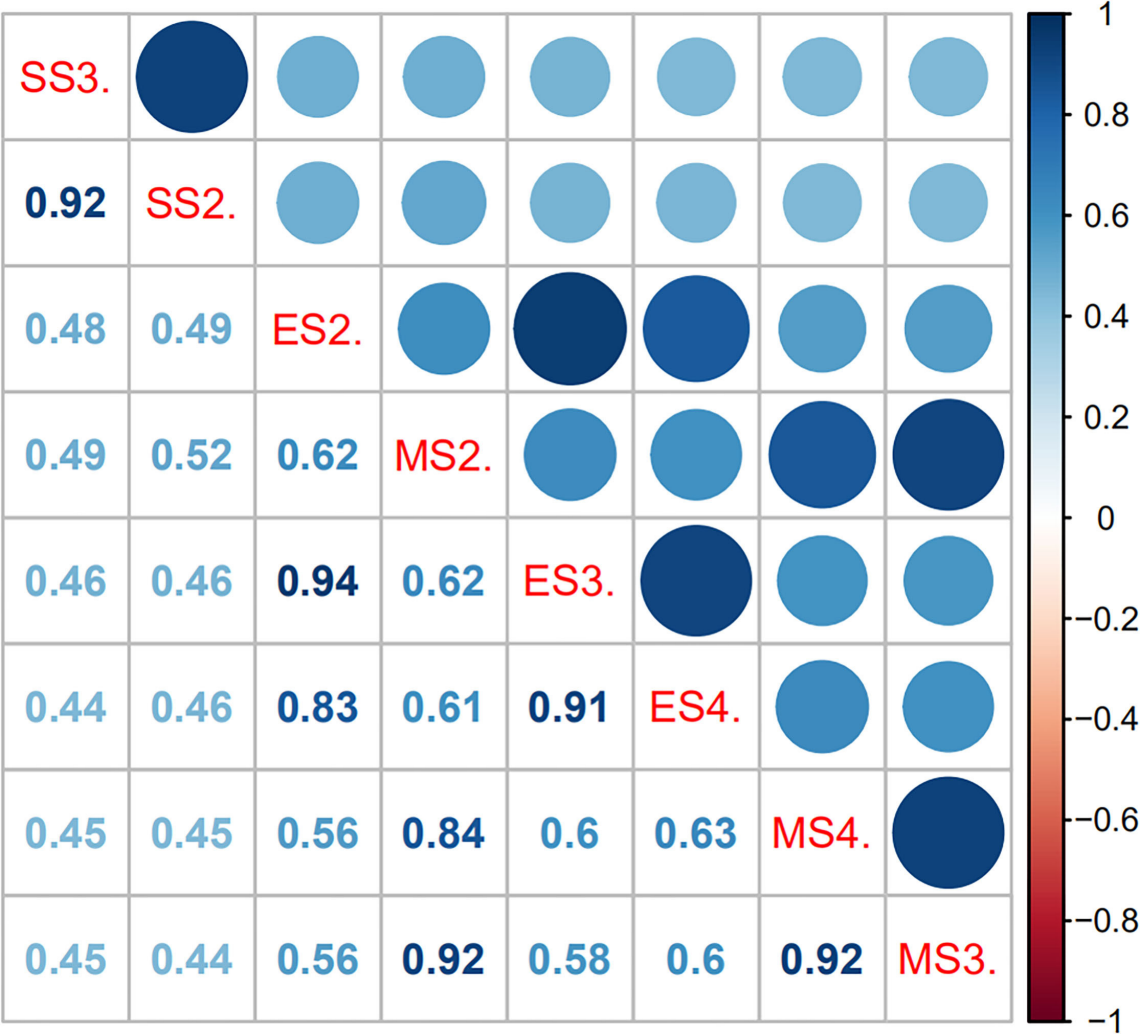
Pearson's correlation matrix for leaf angle in sugarcane different growth stages. The shaded scale refers to the strength of correlation. In Pearson's correlation, abs *r* = 0.5–1 means a greater correlation, abs *r* = 0.3–0.5 means medium correlation, abs *r* = 0.1–0.3 means lesser correlation, and abs *r* = 0–0.1 means no correlation. SS2. and SS3. denote the +2 and +3 leaf of the sugarcane seedling stage, respectively; ES2., ES3., and ES3. denote the +2, +3, and +4 leaf of the sugarcane elongation stage, respectively; MS2., MS3., and MS4. denote the +2, +3, and +4 leaf of the sugarcane mature stage, respectively.

The leaf angle varied greatly among different sugarcane accessions in the panel of 216 sugarcane core parents and their derived lines (Figure 2 and Supplementary Table 1). Leaf angle varied from 12.72 to 58.20° in different stages. The average angle of +2 leaves in the seedling stage, elongation stage, and mature stage was 28.69, 20.21, and 21.82°, respectively, while the average angle of +3 leaves was 35.12, 25.36, and 25.86°, respectively, and the average angle of +4 leaves at the elongation stage and mature stage was 31.44 and 30.44°, respectively. Although the average angle at the seedling stage was the widest, the coefficient of variation was low, which indicated that there was little difference in leaf angle among different accessions at the seedling stage. With the growth of sugarcane, the difference in leaf angle became wider, until it reached its widest in the mature stage. The dispersion of leaf angle also increased as the growth of sugarcane with a variation coefficient ranging from 13.78 to 23.78. Skewness and kurtosis ranged from 0.49 to 1.16 and from 2.82 to 4.70, respectively (Table 1). As shown in Figure 2, the frequency distribution was a continuous normal distribution or skewed distribution, indicating that a sugarcane leaf angle is a quantitative trait controlled by multiple genes. Pearson's correlation analysis showed that there was a significant positive correlation between leaf angle in each growth stage with a correlation coefficient of 0.44–0.63 and a high positive correlation among leaves in the same stage with a correlation coefficient of 0.83–0.94 (Figure 3). The heritability of the leaf angle was 0.7966, 0.7923, 0.8070, 0.8471, 0.8195, 0.8721, 0.8954, and 0.8924 in +2 and +3 leaf angle in the seedling stage, +2, +3, and +4 leaf angle in the elongation stage, and +2, +3, and +4 leaf angle in the mature stage, respectively.

**Table 1:** Variation of sugarcane leaf angle at different growth stages in the panel of 216 sugarcane core parents and their derived lines.

**Stage**	**Leaf**	**Minimum (**°**)**	**Maximum (**°**)**	**Mean (**°**)**	**Standard deviation (**°**)**	**Coefficient of Variation (%)**	**Skewness**	**Kurtosis**
Seedling	+2	20.55	40.07	28.69^a^	3.98	13.87	0.49	2.82
	+3	25.21	50.18	35.12^a^	4.84	13.78	0.63	3.15
	+2	12.72	32.58	20.21^c^	3.27	16.18	0.53	3.67
Elongation	+3	16.54	43.59	25.36^b^	4.22	16.64	0.78	4.58
	+4	21.88	46.48	31.44^a^	4.65	14.79	0.77	3.86
	+2	13.08	38.33	21.82^b^	4.85	22.23	0.82	3.44
Mature	+3	14.49	50.51	25.86^b^	6.15	23.78	1.16	4.7
	+4	15.86	58.2	30.44^a^	7.03	23.09	0.93	4.13

a, b, c *indicate means are significantly different (P < 0.05)*.

### SNP Markers and Population Structure of the Natural Population

**Figure 4:**
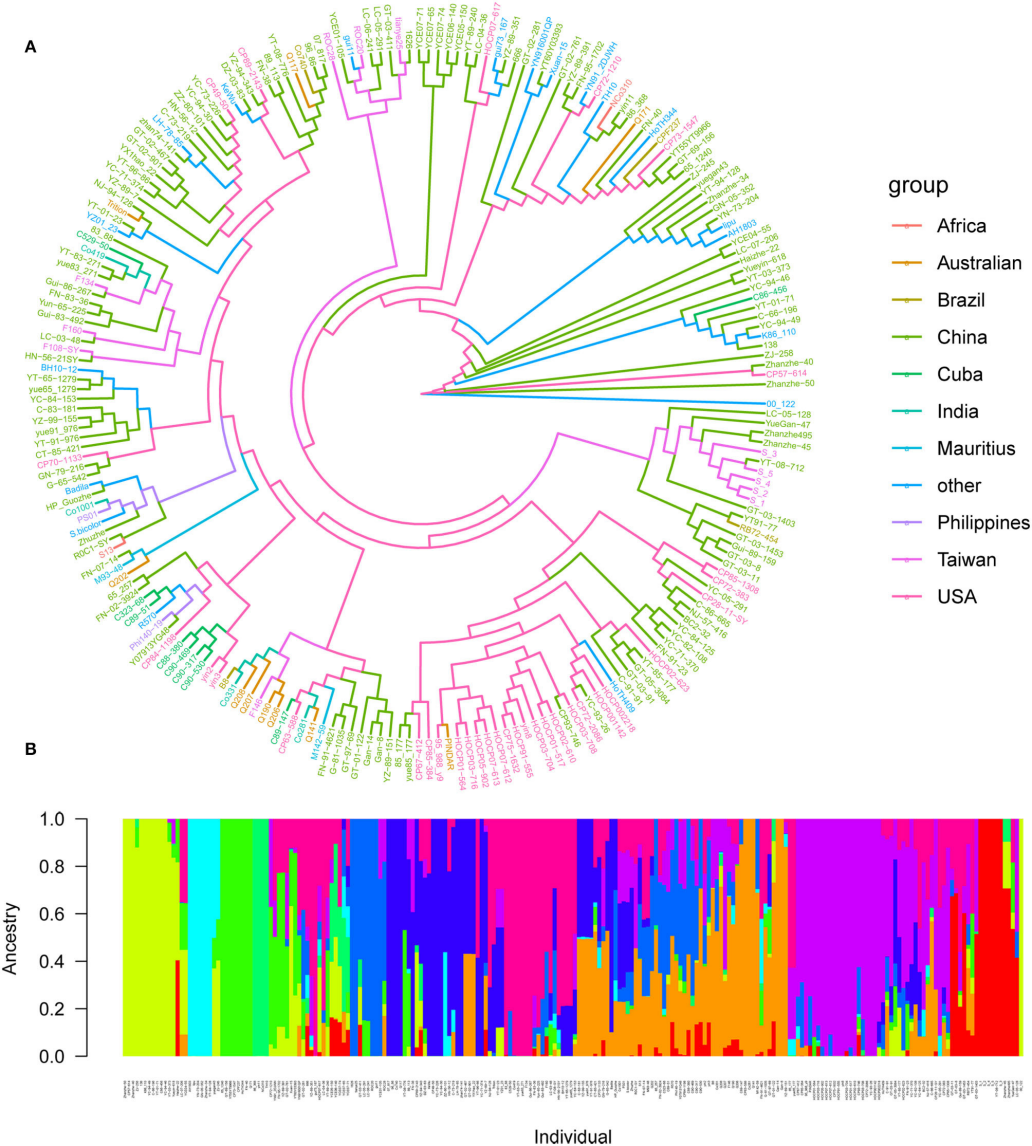
Population structure of 216 sugarcane core parents and their derived lines. **(A)** Phylogenetic tree of 216 sugarcane core parents and their derived lines. Each distinct group was marked with a specific color; **(B)** subpopulations inferred using structure.

A total of 4,584,312 SNPs were obtained following filtration and screening with Plink. Among them, 269,523 SNPs (5.88%) were located within a gene, 144,189 SNPs (3.14%) were situated upstream of a gene, while 143,302 SNPs (3.12%) were situated downstream of a gene, and the remaining 4,027,298 SNPs (87.86%) were located in intergenic regions. Based on the 4,027,298 high-quality SNP markers, 216 core parents and their derivatives were divided into 10 subgroups according to the best *K*-value, and they were Africa (2 varieties), Australia (10 varieties), Brazil (4 varieties), China (125 varieties), Cuba (9 varieties), India (4 varieties), Mauritius (2 varieties), Other (19 varieties), Philippines (2 varieties), Taiwan (12 varieties), and USA (33 varieties), respectively, suggesting that our panel may originate from the admixture of 10 populations (Figure 4). Whole-genome SNP markers were initially used to analyze the LD level of sugarcane leaf angle at different growth stages. The correlation coefficient (*r*^2^) was >0.1, and when *r*^2^ was 0.10, the LD decay rate was 10 kb.

### Genome-Wide Association Study of Leaf Angle

**Figure 5:**
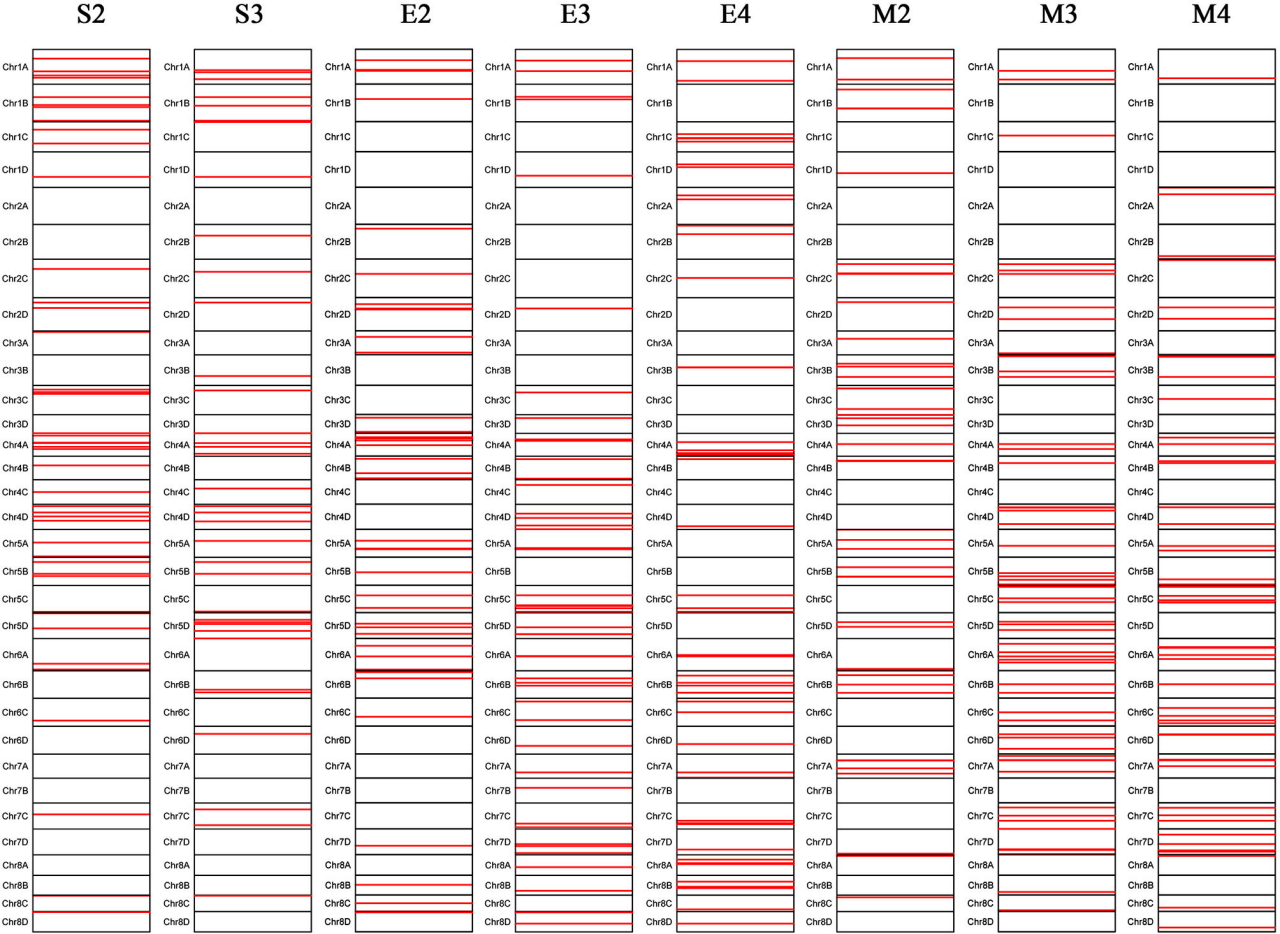
Chromosomal locations of the leaf angle loci mapped in sugarcane three growth stages. S2 and S3 denote the +2 and +3 leaf of the sugarcane seedling stage, respectively; E2, E3, and E4 denote the +2, +3, and +4 leaf of the sugarcane elongation stage, respectively; M2, M3, and M4 denote the +2, +3, and +4 leaf of the sugarcane mature stage, respectively.

**Figure 6:**
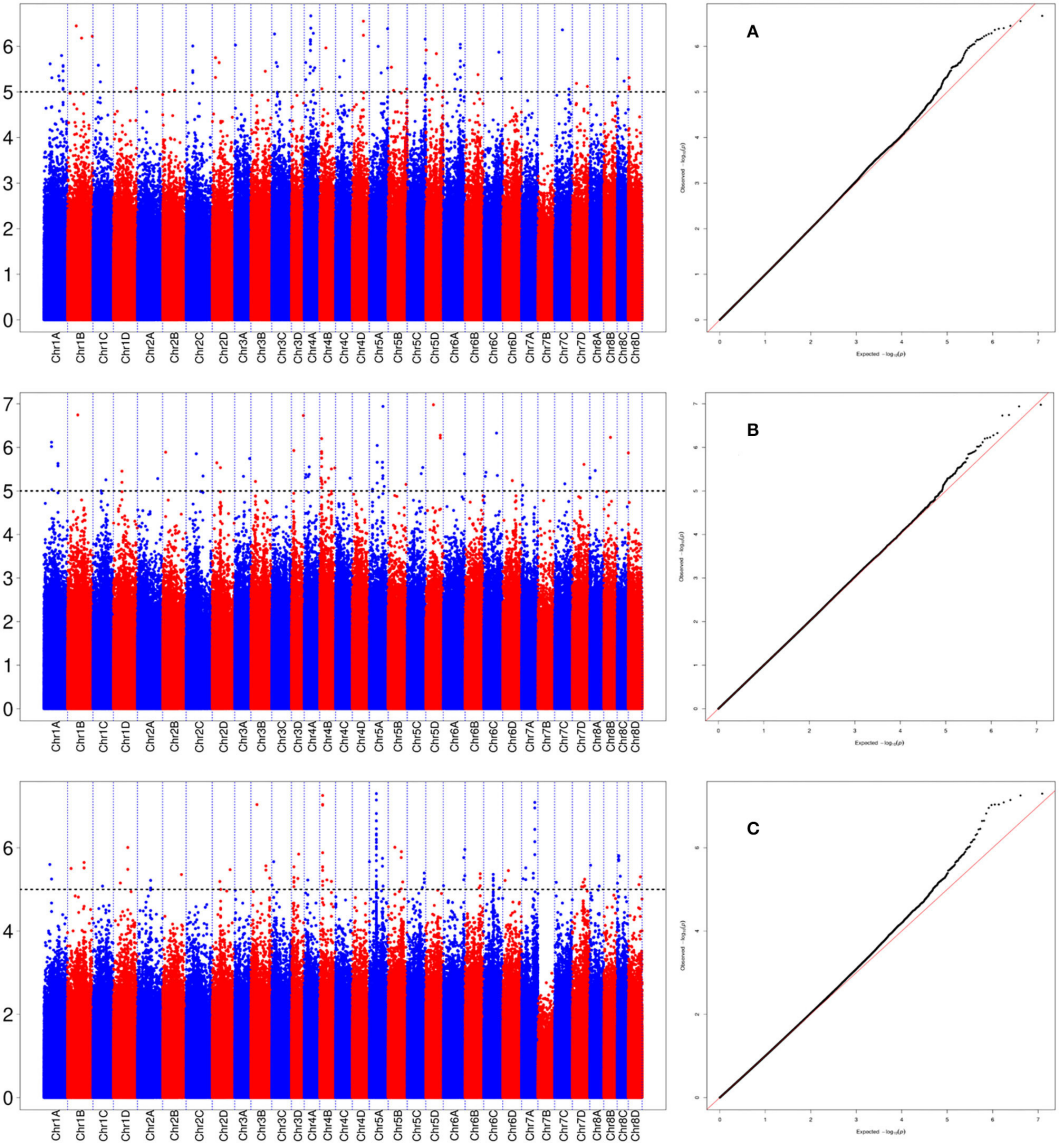
Manhattan plot for the locus of Ss6A_102766953 in sugarcane three growth stages. **(A)** Denotes the +2 leaf of the sugarcane seedling stage, **(B)** denotes the +2 leaf of the sugarcane elongation stage, and **(C)** denotes the +2 leaf of the sugarcane mature stage.

A total of 288 SNP loci were found to be significantly associated (*P* <0.001) with eight leaf angle phenotypes for two repeats (Figure 5 and Supplementary Table 3). There were 69, 113, and 119 loci detected in the seedling, elongation, and mature stages, respectively. One locus (Ss6A_102766953) was mapped in chromosome 6A near the SNP marker of Chr21_102766953 by the phenotype of all these three stages synchronously (Figure 6 and Table 2). Eleven loci were detected by two of these three stages, they were *Ss1A_ 68079563, Ss1A_ 70216053, Ss1A_ 71163098, Ss4A_ 51223571, Ss5A_ 63761550, Ss5C_ 86542573, Ss5D_ 30890024, Ss6A_ 53870145, Ss6C_ 46060170, Ss7C_ 58432083*, and *Ss7D_ 67376640*, accounting for 1.48–4.90% of the phenotypic variation (Table 2), and they were considered to be the elite alleles in this study. Furthermore, 14 loci were identified by the +2 and +3 leaves in the seedling stage, while there were three loci detected in the +2, +3, and +4 leaves in the elongation and mature stages, respectively (Supplementary Table 3).

**Table 2:** List of candidate genes associated with elite loci of sugarcane leaf angle.

**SNP tag**	**Candidate gene**	***E*-value^a^**	**PVE(%)^b^**	**Functional annotation**
Ss1A_ 68079563	Sspon.01G0018220-1A	2.35E-06	2.92	Sulfate transporter
Ss1A_ 70216053	Sspon.01G0018870-1A	3.00E-06	1.74	Ubiquitin-conjugating enzyme
Ss1A_ 71163098	Sspon.01G0019040-1A	2.49E-06	2.21	Auxin response factor
Ss4A_ 51223571	Sspon.05G0009970-2D	1.32E-06	1.84	amino acid transporter ANTL1-likeG
Ss5A_ 63761550	Sspon.05G0015920-1A	1.32E-06	1.48	Serine/threonine-protein kinase
Ss5C_ 86542573	Sspon.05G0036390-1C	4.54E-07	1.49	Transcription factor MYB82-like isoform X1
Ss5D_ 30890024	Sspon.05G0009970-2D	1.28E-06	4.78	amino acid transporter ANTL1-like
Ss6A_ 53870145	Sspon.06G0009970-1A	1.35E-06	4.90	Glucose-6-phosphate/phosphate-translocator precursor
Ss6A_102766953	Sspon.06G0018820-1A	1.43E-06	1.67	MYB transcription factor
Ss6C_ 46060170	Sspon.06G0010540-2C	2.24E-06	2.86	Serine-rich protein-related
Ss7C_ 58432083	Sspon.07G0033260-1C	7.95E-07	2.78	Thylakoid membrane protein TERC chloroplastic
Ss7D_ 67815942	Sspon.07G0034190-2D	1.55E-06	3.13	Probable serine/threonine-protein kinase

a *Indicates the significantly associated SNP sequences blast with transcriptome library of sugarcane based on E-value <10^−5^.*

b *Indicates the phenotypic variation explained by the mean value of the individual locus detected in different stages*.

### InDel Markers Linked to SNP

An InDel site linked to SNP Ss6A_102766953-G-C was obtained by sequencing 12 amplified products from 10 core parents and their derivatives. The InDel marker was closely linked to SNP Ss6A_102766953-G-C, which was 18 bp nucleotide sequences. The nucleotide sequence was TCCAGTTGTATTTGTACC. When the SNP site was C, the amplified sequences were 218 bp. When the SNP site was G, the amplified sequences were 200 bp. Ss6A_24693233-G-C was a significant SNP greater than the threshold in genome-wide association analysis of +2 leaves in the seedling stage, +2 leaves in the elongation stage, and +2 leaves in the mature stage.

### Candidate Gene Analysis

Candidate genes were searched in the range of LD decay distance (500 kb) of SNP upstream and downstream of –log10 (*P*-value) within each locus. According to the annotation of gene function and its expression position/expression level in the reference genome, the most likely candidate gene was selected as the candidate gene of this site. A total of 5,571 candidate genes were located in these 288 loci, of which 3,892 had GO functional annotations (Supplementary Table 3). These candidate genes are mainly related to brassinosteroid LRR receptor kinase precursor, auxin response factor, gibberellin receptor, chloroplastic, auxin synthesis/signal transduction, serine/threonine protein kinase, and various transcription factors. They are directly or indirectly associated with leaf angle.

### Core Parents and Their Derivatives With Narrow Leaf Angle Tagged With SNPs

A total of 13 germplasm resources carrying a different combination of elite loci and narrow leaf angle (<30°) in all these stages were selected as given in Table 3. Marker heterozygosity remained in all the core parents and their derivatives, except for Xuan-15, such as #1626 at Snp8 (*Ss6A_102766953*) and Snp12 (*Ss7D_67815942*), CP57-614 at Snp7 (*Ss5D_ 30890024*) and Snp9 (*Ss6A_ 53870145*), and CP89-2143 at Snp5 (*Ss5A_ 63761550*) and Snp9 (*Ss6A_ 53870145*). This suggested that these materials can also be used for fine mapping of these loci. These 13 core parents and their derivatives with their leaf angle and nearest marker should be useful for improving sugarcane leaf angle *via* MAS.

**Table 3:** The elite core parents and their derivatives with narrow leaf angle and the nearest markers.

**Name**	**S2+**	**S3+**	**E2+**	**E3+**	**E4+**	**M2+**	**M3+**	**M4+**	**Snp1**	**Snp2**	**Snp3**	**Snp4**	**Snp5**	**Snp6**	**Snp7**	**Snp8**	**Snp9**	**Snp10**	**Snp11**	**Snp12^*a*^**
1626	25.29	29.01	15.39	20.51	28.55	13.95	17.80	20.78	T	A	C	G	C	C	G	C	C/T	A	A	C/T
CP57-614	22.01	29.55	14.41	17.09	22.59	15.65	16.92	20.00	T	G	C	T	C	C	G	G/C^b^	C	A/G^b^	A	C
CP89-2143	24.62	28.29	18.75	22.29	28.84	18.17	20.59	24.57	T	G	C	T	C/T^b^	T	A	C	C	A/G	G	C
GT-03-411	24.22	29.88	18.24	22.27	25.23	18.47	21.25	25.35	T	G	C	T/G^b^	C	C	G	C	C	A	G	C/T
HOCP03-708	21.66	29.06	14.89	19.10	27.59	17.00	20.71	23.56	T	A	C	T	C	C	G	C	T	A	G	C/T
Liucheng05-291	21.48	28.42	17.75	22.00	26.73	15.49	18.79	20.80	T	G/A^b^	C	T	C	C	G	C	C	A	G	C/T
Liucheng06-241	22.77	27.88	17.82	23.06	27.83	13.08	14.49	15.86	T	G/A	C	T/G	C	C	G	C	C	A	G	C
Neijiang57-416	20.91	25.35	18.33	23.02	28.45	17.27	20.86	26.39	T	G	C	T	C	C	G	C	C	A/G	G	C
Xuan-15	24.12	28.19	14.98	18.53	25.61	19.05	24.16	24.66	T	G	C	T	C	C	G	C	C	G	G	C
YCE07-71	23.04	27.49	15.27	19.84	27.25	21.42	24.16	29.96	T	A	C	T	C	C	G	C	C	A/G	G	T
YT-01-71	22.75	28.54	15.99	20.84	24.81	18.92	20.74	23.14	T	G	C	T	C	C	G	G	C	A/G	G	C
Zhanzhe-40	25.11	28.88	12.72	16.54	21.88	15.13	16.55	19.09	T	G	C	T	C	C	G	G/C	C	A/G	G	C
Zhanzhe-50	20.55	26.89	16.17	19.45	22.72	16.39	18.08	20.54	T	G	C	T	C	C	G	C	C	A/G	G/A	C

a *S2+ and S3+ denotes leaf angle of +2, +3 in seedling stage, E2+, E3+, E4+, M2+, M3+ and M4+denotes leaf angle of +2, +3, and +4 in elongation and mature stage, respectively. Snp1 to Snp12 were the nearest markers of Ss1A_ 68079563, Ss1A_ 70216053, Ss1A_ 71163098, Ss4A_ 51223571, Ss5A_ 63761550, Ss5C_ 86542573, Ss5D_ 30890024, Ss6A_102766953, Ss6A_ 53870145, Ss6C_ 46060170, Ss7C_ 58432083, Ss7D_ 67815942, respectively.*

b *Indicates heterozygous*.

## Discussion

The leaf angle of sugarcane is an important factor in determining plant architecture. Compact plant architecture can improve photosynthetic efficiency through reasonable close planting to enhance the yield of sugarcane per unit field area (Luo et al., 2004, 2013). However, the research related to sugarcane plant architecture and leaf angle is focused on epigenetics, which may be due to the polymorphism, high chromosome numbers, and large complex genome size of sugarcane. To study leaf angle deeply, it is necessary to find out a gene/QTL that determines leaf angle in sugarcane. In this study, 288 SNP loci were found to be significantly associated with leaf angle at the seedling, elongation, and mature stages by GWAS. A total of 69, 113, and 119 loci were detected at the seedling, elongation, and mature stages, respectively. This might be related to the variation in leaf angle at each stage. The average leaf angle at the seedling stage was 31.91°, but the coefficient of variation was low among all the materials. The leaf angle at the elongation and mature stages was 26.04 and 25.67° with a wider variation, respectively. Moreover, Pearson's correlation analysis showed that there was a significant positive correlation between leaf angle at each stage, especially for different leaves at the same stage, which was consistent with the GWAS mapping results. Among them, one QTL was identified at all three stages synchronously, while the other 11 loci were detected at two stages, indicating that these QTLs were consistent QTL across development stages. This is consistent with the conclusions obtained in rice by Hittalmani et al. (2003) and Xu et al. (2015). In addition, many loci were not consistent across different leaves and stages, which might be due to the influence of different growth stages and environmental factors.

In this study, 12 consistent loci that determine sugarcane leaf angle were discovered. *Ss6A_102766953* was stably identified from all three stages, and the candidate genes within this locus indicated that MYB transcription factors might play a role in determining leaf angle. The MYB transcription factor family is one of the largest transcription factor families in plants and is involved in the developmental process of plant cell differentiation, morphology, etc. Dubos et al. (2010) found that MYB transcription factors regulate Arabidopsis growth and development, auxin response, primary and secondary metabolism, cell fate determination, plant growth and development, and responses to various biotic and abiotic stresses. Zhang et al. (2007) and Shin et al. (2007) found that MYBs are induced by ABA, IAA, CTK, GA, ethylene, and other plant hormones, indicating that MYB transcription factor genes in plants are widely involved in the responses to plant hormones. Cao et al. (2015) found that OS JAMyb encoding the 2R-MYB protein is expressed in root, stem, leaf, leaf sheath, panicle, and other parts of rice. Therefore, it is suggested that this candidate gene might be associated with leaf angle and can be further studied in future.

Auxin is an important signaling molecule and regulates the growth and development of plants, such as promoting cell elongation, vascular differentiation, and regulating the size of leaf angle. In this research, *Ss1A_ 71163098* was detected in the seedling and elongation stages, and the candidate genes showed that this locus contains an auxin response factor. Previously, Moon et al. (2013) found that auxin accumulated at the boundary between the leaf and sheath through fluorescence imaging of the auxin-directed transport protein ZmPIN1a, indicating that auxin is involved in the positional initiation of the leaf sheath. Zhang et al. (2014) identified a gene that controls leaf angle, *LAZY1*, on maize chromosome 4 *via* map-based cloning, which showed the change in leaf angle caused by auxin effects on cell development. The auxin-related gene *FIB* identified in rice is homologous to the auxin biosynthesis gene *TAA* in Arabidopsis, encoding tryptophan aminotransferase. The functional deletion mutants of *FIB* showed smaller leaves and larger leaf angles (Yoshikawa et al., 2014). *Lr47* affects auxin signal transduction by inhibiting the interaction between C-22-hydroxylation and 6-deoxybrassinolide and controls the curvature of the pulvinus, resulting in larger leaf angles and oblique leaf elongation (Miao et al., 2016). The elongation of leaf occipital cells in *LC1* mutant plants is affected by auxin and has an increased leaf angle phenotype (Zhao et al., 2013). The F-box protein TIR1 regulates the angle of rice leaves by binding IAA and Aux/IAA, which leads to ubiquitination, degradation, and release of ARF transcription activity. Overexpression of *OsIAA1*, which encodes Aux/IAA protein, reduced the inhibition of auxin treatment on root elongation but increased the sensitivity to 24-epibrassinolide in rice. Overexpression of *OsIAA1* resulted in significant morphological changes such as decreased plant height and increased leaf angle (Song et al., 2009). In addition, IAA can also participate in the regulation of rice leaf angle through interaction with BR. IAA is involved in the OsBRI1*-*mediated BR signal transduction pathway. OsARF11 and OsARF19 bind and stimulate the promoter of *OsBRI1* to induce changes in leaf angle in rice (Shen et al., 2010; Zhang et al., 2015).

Some functional kinases, such as serine/threonine protein kinase, are also the main factors regulating leaf angle. *Ss5A_ 63761550* was identified in the elongation and mature stages, and the candidate genes showed that this locus is associated with serine/threonine protein kinase. ILA1 is a functional kinase with serine/threonine protein kinase activity, which mainly exists in the nucleus and expresses in the vascular bundles of the leaf pillow. It affects the leaf angle by regulating the formation of mechanical tissue and the abnormality of the cell wall composition of the rice leaf pillow. The T-DNA insertion mutant *ila1* showed the character of increased leaf angle. Through the anatomical analysis of *ila1* mutant, it was found that the number of vascular bundles in the leaf pillow decreased and the number of thick-walled cells decreased. Moreover, the mechanical tissue abnormality of the mutant leaf pillow led to the lower content of cellulose and other cell wall monosaccharides, which led to the poor mechanical support of the mutant and the increased leaf angle (Ning et al., 2011).

Many other elite loci mapped in this study are also very important in regulating the leaf angle. The candidate gene *Ss1A_ 68079563* is predicted to be a sulfate transporter (H^+^/SO${}_{2-}^{4}$ cotransporter). Their transport function depends on the membrane potential gradient maintained by an H^+^ pump, and the sulfate transporter is higher in mature or older leaves (Hopkins et al., 2004). The candidate gene *Ss1A_ 70216053* binds ubiquitin-conjugating enzymes that are mainly involved in the ubiquitin–proteasome system (Gagne et al., 2002), regulating ethylene, GA, IAA, and other hormone signal transduction (Qiao et al., 2009; Hirano et al., 2010; Zhao et al., 2010; Miao et al., 2016), thereby indirectly affecting the leaf angle. The candidate genes *Ss4A_ 51223571* and *Ss5D_ 308900244* are associated with amino acid transport that is necessary for the growth and development of plants (Hammes et al., 2010). The candidate gene *Ss6A_ 53870145* is related to the glucose-6-phosphate/phosphate translocator precursor that is preferentially expressed in non-green tissues and mediates the transport of glucose-6-phosphate (Glc-6-P), triose phosphate, and glycerol-3-phosphate (3-PGA). Plastids of non-green tissues are the main storage sites of carbohydrates as starch in heterotrophic tissues. Through GPTs, non-green plastids can transfer sugar from the cytoplasm into carbon sources in the form of Glc-6-P to drive the synthesis of important substances such as fatty acids, amino acids, and starch, thus providing precursors for the pentose phosphate pathway (OPPP; Kunz et al., 2010). The candidate gene *Ss7C_ 58432083* associates with the thylakoid membrane protein TERC in the chloroplast and plays an active role in protein transport, photosystem assembly, and thylakoid membrane stability (Dekkera and Boekema, 2005). The candidate genes of these excellent SNPs are directly or indirectly related to leaf angle and should be further investigated in future.

An InDel marker closely linked to SNP site was obtained in this study. The InDel markers can be detected by amplifying the target fragment, which can predict the sugarcane plant type in a specific stage. The results can be used for molecular marker-assisted breeding to accelerate the breeding progress of sugarcane leaf angle and improve the selection efficiency.

## Conclusion

In summary, a total of 288 SNP loci that contribute to leaf angle were identified by GWAS at the seedling, elongation, and mature stages of development in sugarcane. Twelve of these SNPs were detected in at least two of three developmental stages. An InDel marker (SNP Ss6A_102766953) linked to narrow leaf angle was obtained. The candidates of these elite loci were analyzed compared to the function of their homologs in rice, corn, Arabidopsis, and other plants in this study. These candidate genes are mainly related to MYB transcription factors, auxin response factors, serine/threonine protein kinases, sulfate transporters, ubiquitin-conjugating enzymes, amino acid transporters, glucose-6-phosphate/phosphate translocator precursors, and the thylakoid membrane protein TERC. Thirteen core parents and their derivatives tagged with SNPs (Table 3) can be used as narrow-leaf-angle donors for MAS.

## Data Availability Statement

The original contributions presented in the study are publicly available. This data can be found at: PRJNA810929.

## Author Contributions

YQ conceived the experiment. XC, ZH, and DF performed the research. JF analyzed the data and modified the manuscript. XZ analyzed the data. JX, BW, YL, and MZ measured the leaf angle and collected the data. XF helped prepare markers for GWAS. XC drafted the manuscript. All authors read and approved the final manuscript.

## Funding

This work was funded by the special project of Guangdong Academy of Sciences, China (2019GDASYL-0103034), the National Key Research and Development Program of China (2018YFD1000503), the National Natural Science Foundation of China (32072027), and the Special Project for Research and Development in Key areas of Guangdong Province (2019B020238001).

## Conflict of Interest

The authors declare that the research was conducted in the absence of any commercial or financial relationships that could be construed as a potential conflict of interest.

## Publisher's Note

All claims expressed in this article are solely those of the authors and do not necessarily represent those of their affiliated organizations, or those of the publisher, the editors and the reviewers. Any product that may be evaluated in this article, or claim that may be made by its manufacturer, is not guaranteed or endorsed by the publisher.

## Supplementary Material

Supplementary Table 1 Leaf angle and corresponding genotypes of sugarcane natural population at different stages.

Supplementary Table 2 Primer sets used in the PCR amplification.

Supplementary Table 3 Candidate gene information for all the significant loci of sugarcane leaf angle in this study.
