# Supplementary material for: Identification of Genetic Loci for Sugarcane Leaf Angle at Different Developmental Stages by Genome-Wide Association Study
Source: Front Plant Sci. 2022 May 27;13:841693. doi: 10.3389/fpls.2022.841693 (PMC9185841; doi:10.3389/fpls.2022.841693)
Supplement: Supplementary file 1 [file Table_4.DOCX]

Supplemental M. The twelve SNP markers and the sequences used to PCR amplification

>SsChr6A:102766653-102767253 G/C

TTACCAAATTTCACGTTTAACATTTCATTACATGTGTGATGCTGCCACCTTTCCTGCACAGCAGAGATGTAAATCTCATCTAAGCCTATGAAAATAAATCCCCTCCCACCCCATGTTGGCAATATCCACTTGGGTAGCGCACCGTCAGGCAATATCCGCTGTAGTAGAGCGACTGGGAACATCATCCTTTTAATCAGTACCATAGGTTCAGAGCAGTGACTAACAATAATGCTAATACCTCTAATTGTATTTGTACCATTAGATTAGTCCGTTTTTTATAATCCATGGTACCACACTCCGTCACCGAGGTATTAAGCTCGGAGCTAACTACCCACGTTTCTGTACGTAAAGGATGACACAGGCGGTGACCCCATAACCCTCCAAGCTGCCGCTGCCCTCGTTCCCCCCAATGGGATCCTGTGCGGTTCCCATCGATGGTGCCGGCGGGCATTCGTGCCTCGCACGCCCAGAGATCTGCCCGCGGGCAAGAACGGCACGAGGCCAGCGCAGGGATAACAGGGTAGTGGTGCTCGGTAGCATGGAGGAGCTCCTGCATGCGCGCGCGCATGCTTAGCGTGGTGGGACGCCAGCTATCCGCAC

>SsChr1A:71162798-71163398 C/T

TGTCTGCGGTGGGTGTGGAGTGGCCAGGCACGGCCGTGACATTGCAGGCAGAGACGGTGCAATTGGGTGCCGTCGTGACACCATAGACAGAGGCGGTGCAATCGGGTGCGGCCAGGATGTCGCAGATGGAGATGGTGTGATCGGAGCCATCACCCACGTAGGCGGTCGTGTTCGTGACCGAATGGATGGAGGAGGAGGCATCTGAGGCGTCCCTATCGGGCGCGGTGATGCGGTAGGCATCCGCGCCCATGCCACCGACCCCCACCACCATTGGAGAGATGGTACCCGAGGAGGTGCTACCGTAGGAGGAGCTGGTGGCACCTAGGGCCGGCGAGGGGTCCTCCCTGGCCCTAATCTAGGTAAGCGGTGACTCACAAGCATAGGAGACCCTAGATTCGGTGGGTTGACTGGTGGAACCCGGAGGTGACGCTCTTCGTCCTTGACGACGCCGCGGAGAAGAGGGAATGGGGGAGCGTCCACACGGGCGTTGAGTCCATGGTTCACGCCCAGAACACTGCGCTGGGTTCACTACACGACGTCATTACCCCGGCTAGCCAGGTATGACATGTCCGTGCTTCCAGCTTTGATTCCCCCCTTTTT

>SsChr4A:51223271-51223871 T/G

GCCTTGCTAGGGCATAGGCTGCTTCGCAGGATGCCCGTCCTATATAGGGTGCTGGCGTAGAGTATGTTGAGGCCGCTGTCGCCGTCCATCCGCACCTTGGTAAGGCGCATAGTGGCCACGATCGGGCTGACCACGAGCAGGTAGCATCCTGGGTGGGGGACACGATCGGGGTGATCCCCCTAGTTGAAGGTGATGGACGTCTGGGACCACCAGAGTCACATCGGGGTGGCGAGGGTGTGGACGGCGTTCACCTCCTGCTCGGTGATGCGGCGCTACTGACGGGACTCGTACGCCTGGGATCCCCAAAGATCATGAGGCAATGATCGGCCTCGAGGAACTCGGCGTCGTCCTCTAGGGCGGCATTCGCTGGTTCGTCGGCCTTCGGGGCTGGCTTTTGGGCCTTGCCCTACTGGCCAAGGGTGCCGCGGATGTAGCCACTAATGGTGGTGCAGTCCTTGAGGAGGTGCTTCGCCTGCCCTCCATGGAGTGTACAAGGGGCTTCGAGCGCCTTCTCGAAGTGTTCGAGGCGCACAGCGCGCCCACGGGGTTGAGGCCTGGCGGCGTAGTCGGCCGCTGCCACCATCTCCTCCTCGCAGTGCT

>SsChr5D:30889724-30890324 A/G

GGACATTACCAGAAGCGCTCTATCGGTGAGGTAGGGTTTTTTCTCAACAAAAGCTTGGTTTTACATAGGCATGCCAGTTACTTTAACAGATTTGAAAAGCAAAAGAGATGATGTAGCTGCCTGGAGCTCCCTGCGTGGGTGGAGATCTCGCCATCGGCTGTCTTTTGCTCTTGTGGTGACGCCACTCCTGTGCTCCAATATCCCTGCTAGCGCCCCGTGAACTAGGGAACCATCAGGTCGTCCTCCTCTCTGAAGTCTGAGGGTTCTAGGAAGTGGTTGCAATTGTCGAGGAAGTGGCCAAGGGCTTCCAGGTGATCGAGCCCTATCATGTTGACGTCATTCCACCAGTCGCATCGGCCAGGGTTGTCATCGTTCTCGCAACGCCTGGAGCCCCATGGTTGGTATCCCCTTGCGAAGGACTCTAGAGGGTCAGCCATAGTCCATGCCATGGCCTTGCTGCGGGCCTTCGCTGTCCGCTTCATCCTTGGCAATGATCTAGCACCGGCTAGCACGTGACCCCTATGTTTGGGTCGATGGCCTCAGAGTGGAGCTACGACGTGGGTCTTTGGGTGCGTGCTTCTGCCGCCACCGTGGCAGCCA

>SsChr6A:53869845-53870445 C/T

GCGGATCTCGCTCCGTGGCCCCTCCCCTCTCCTTCCTCCGGCAGATTTTGACGGAGGCTTCCCCGTGGCGGCGAGGCCTCCCCGGCAGCGAGGGGCTCCCGGTCGCGTGCTCCCTCTGTTGTGGTGCGACGAGCTTTGGCGGCAAGCGGAGGAAGGAGATCGGCCGCGGACGACAAGAGTTGCGGCAGACGGAGGTGCGGAGCTCGACCCCTGGGGAGGAGGCGGCCGCAGGGGAGGAGGCGAGCTCGACCGCTGGAAGGAGGAGCTCGGCCGCTGGAGAGGAGAGGCGAGCTCGTCCGCGAGGGAGGAGGCGGCAGCGGGCGGAGGTGTGAGCTTGGCTGCGCATGGATCGGGAGGAACCGAGGAACAAGACCCGACCTTATCCATTGAACGCGAAGAAAAGAGAAAGAGGGAGAGTCCCACTGATAGGCAGGCCCCACGGTGTCAGTGGCTATGGGAAGAAATTTGGGGGCCTTTATTTGGGGGCTACTGCTGGAGTGCAAACACAAATTTGGACCAACAAAATTAGAGGTGGGCACCCAAATCGAAAATAAGGGGTCTTGATTTATGGGCTACTGCTGGAGTTGCTCTGAGTCAGCA

>SsChr7C:58431783-58432383 G/A

CTCAGCAGTACGCGGGCGCCATTCACTCCGATGACTTGTTCCTGTCTCCCGTCCGCGTTCCGAAGGCCGGCGCGTCGCGCAGCCTCAACTTTAGGCCGCCTCGGCCCGGCGGCCGCGCTGGAGGATCTGGATCTGCTACGTTCGCCATGCATTCTCCGTCTGATGGAGGTGGCTACGTCGACGCTCCACCTCCTGAAGGGTTCGTCGCAAACTTTGGTTCAACCCGTTCCAGGGGCCGCGCCCCACGTGCTCGTCGGCGGGGGCCTACCATTCACGCAGATAACTTCGAGGTACACCCTGCTGCATTCTACTGTTTTTAGTTCGGGGTTTTGCTTTTGCTGTTGCCTCATATAACCATGCGAATACTGCCTTTTGCTATTGCCTGATACAACCATGAATATTGTCGTTTGTTTTCTGTATTTGGTGCTCTGCCTCTTCAACGTTTAGGTGCAGCATAACAAAGCATGCTGGAGCCTAGAAAATGTGGAGCACTACTATGACGCCTACATCGAAGAGATGAGGAAAGGGAATGCTCCTGCTGGACAAATGAGCAAATGGGGTTGGTCCAACCTGCAATCCAGTTACTACGAGATCAGTGGT

>SsChr7D:67815642-67816242 C/T

CAAAAAGCTGTTCCTAATTTTTATTCTGACATGTAGGGCTCACGTATCGCCCCCGCCCCACAAGGCCACGACAGGATAGTTTTCTAAAGCTCGATGCCTTGAAAGGATTTAAACCCGGATAATGGAATCCGGACATCCATTCAGTATTTGTACCGTATCAAAATCAAAATACCTAAGAGCAAGTACAGTAAAGGGCTACTGGTGGACCGTATGCTGAGGTGGAGAGAGGGAAGGAGAGAAGCGGGCTCTAAGTGCTGACTTACGAGAACCAAGGAAATTTGTGCGAGAGATAAGTGGAACATGCATTAATAGTGAAGATATAAATACTATATAGGTGGGCTGAGAGGTAGGCTACAAGAATTAATACAACCACTAGCCGGTTGTATTATTAGCCTTTTCTAATATTGGATTTCTAATACATACATGATATAGATATCAATATCCAACGCATTCGATACTATAGGTGTGTTTGGTTAGGGGCTAAGCTTGCCTAATGCACCCCAGCCATGTTTGGTTGGTTGCCCAGGCCATTCAGCCAGGCTTAGGTAAGCCAAAAGGGAGCAGCTAGCCAGACTTAGCGAAAATGGCGGGGATGCTCAT

>SsChr1A:68079263-68079863 T/C

TCTTTATTGGGTAATACCCATCGGGTACTCGGGTTTCGGGTACCCGTTGCCATCTCTAGCCACGAGGTTGGCGTAGGTGCTCGTGGCATTGCGTAACCCGTAAGGCCACGCCTGCTGTAAGGGTGTGGAGATCAACTACGGGGAGAGCAGAGGTCCCCTGGTGAGTTGTGACATAGTGTTCATGAAGGTGAAGTAGAGGAGGTGGAGATCGTCCTAGGACGGGTTCGGTCCCAGGAGAGCGTCGAGCTCGCGCTCCAACTTTGGGTAGTCACCGTCGCGGGGCTGGACGCCTCTGGAAGTCCGTGCCCTGGAGGTGTGCTGGCTAAGGCCAGGCATCGATTCGATGATAGAATCGATGCCAGCGGATGGTCCAGAGGGTCTAGAGATGGAGGGCAGGGCGCGCCCCCCTTCCGTGCTGGCGAAGTTCGGCTCCCTGGTGCGCACGTCGAAGCCAGGAGGCGACGTAGGATCCACTGGAGCGGAGGGAGGGAGCAGAACTAGGTCGTGGTCTACTCCCGTGTAGACAAAGTCCGGGCTCCCAAAACGGATGTGGGAGCCCAGAGTGAAGCAGATGCGAGGGTCAGCCTTCCTAGGGGAGTG

>SsChr1A:70215753-70216353 G/A

CCGCCGTCGATGCCCTCCAGCGTGTGGTAGTGGCAGCCCGCGGTGGCCCCGAAAAAAGGGTTCGTGTTCGCATCCGCCTCCCTCCCCCTCACTCCTCCACCACCCCTCCTCTTCCTCTTCAAAACGAAGATTTGAGCGGCCAACCAAGCGGATCCTAGGCCAGATCTAGCGTCTACAACCCGCGGCCGCCTACCCGCCTTGGCACTCTGGCTCCAGTCTGGGTGCCGGCCATGAAGCTCGCCATCGATGGTTCAGGTGCCCCTTCTTCATCCTATGTGATGAAGAAGAAACCGCGTCGCGGGCTCCTCAATGGTAGATGGTGGTGGCCAGGTCTGGGAAGCTAAGGCCGGCGGCCGAATCCGATCTAGCCAGGCCCTAGCTAGCTGAGGCTCGTGTGCCTAGATCTGCACCCTTCGAGGGTGGCAGCGGCTGGCCAGGGCCGGACTCGGCAATAGCCTTGCTGAGCCACGCGCCTACCCACGCCTGCCGTTGGCTAGCGGCCAAGGCTGTGGCTAGTCCTGTCCTACATCAGCCAGCTCTGGTGCACATGCCGACGGCCTTGCTAGGCCACACATCTACCCACGCCTACCGGTGGCTGGC

>SsChr5A:63761250-63761850 C/T

GTAGCGCTAGGACAGTGGTTCTTTAGACTATTCGCACTCATGAACCTCTATATTCGTCCTCTATACATCCTAGTCATCAAGATTCTCTCCTCTATACCCAATTCTCCCGCACTCATACATTCATTATATCATCCTCTATCCACTGTGGGGCCCACTTGTCACTTTATCTAAATATATTCATGTGAGATCTTGTTTTGAAGAATTTATTGAAACAAATTTAATGGTACAATCGAAATTTAATTTGGTGTTTTGGTTTGTGAGTTATAACATTTTGTTGCAAGTTGAAACTATGGGATCTGCATGCGCAGTGTTGGACGGCGCTCTCTCTCCCTTCCTTCTGTCTAGCGCAGCAGTCGTGGGCCCCGCGCCATGCAGGTAGCGGACGAACGGCATCGCGCTGCAAATGGCGGACGGCGCGCGATAGCGTCTGAGGGTGGAGTACCTGCTGCATGCCATTTTCACGGTAAATGGAACGGTAAGATGAATAGCGCACCGGGATAGCGTACCCAGTGCAGCTAGTCTTACACTCAGCTGCCCAGGCCGCCAGGCCCAGCAGAGGCATTCAACTATTCAAGTCTTCGGGGGTATGATACATCATGT

>SsChr5C:86542273-86542873 C/T

CCCACCGAGGACGTCTCGTAGCGTCCGGGCAGGTAAGATTTTACACTCTCATATCACATATTTCGAATTATAAGTTCATATATGTGTGTCAAGTAGCATTTGTAATTCGTTTAAATGCAGAACCTTCCTCTCCTTCGTTCTAGAGTACATGGAGGGTTCTTGGAAATGTAGTACGACGAGAGGTACACACCGTACCTATCGAGAGCTGGCCTAGACGTCGTATCTTTCCAGGTTCGACGTGGCCTGCCTTTGTTCAACTCGGCGGACTTGACTGCGTTGGTTGACAGGTATTTTTTTAACAAATAGGTTGTATTAATAATCATTTGTTCATGCGGATATGTTTCTAAGAACCAGTTTTGTTTTGCTAAAATTATAGGTGGCGACCTGAGACTCACAGCTTTCACATGGCGTTTGGAGAGATGACAGTCACGCTACAGGACTGTCAGAAGATGCTCGGTCTGAGTATTCGTGGGAGGGCAGTGACAGGGCTGTGTAGGTCAGAGGGTTGGAGAGCTAGGGTGGAGGCCTTCCTTGGGCGTGAGGTGGGTGACCAAGGAGAGCGCACTTCTGGTGTCCTTCTCTCCTGGCTTAGGGCTGAGT

>SsChr6C:46059870-46060470 A/G

TTTTTATCTGTCATCTGTGATCCTAGAACTGAATGTAAAGGCTCATAATTGCCGCTTGTTTATTTCAATGAGTACTATTAATTGCATGCTTTAATAAATGTTGCAATAACAGACAATATGCTGCATGAAAAGTAGACTTCTTTTTGTCATATTATAGCAATTTCACTAACCTCTGGGGCTAGAACTTCGACTTACAACAAATTTAATCCTGGACATATACCAAGCTGGATCATGGAACCAACGAGTTAAGAGAGTGTATGGCCACCTGGAATCCAGAAATTGCAGTAGATAAACAATATAAGAACATACCTCTGTGATATCTGAAAAGAAGCCAGTAACATCAGCCACACGCAGTGATCTTCCTTTGCTGTGGATTCTTAGTTTCTCCACATCCATAACAAAAGCTCTTCCAATAACAAAAGAAAAACATGTCCTTCTACTAACCTGTAGAAATTATGAAGGAAAGAAACAGAAGGCCATTATTCAGTTTTCTAAAATAGAGAGTAGACATGAACTCATTGAAATAAATGCAAAGTAACAGCATAATTTATTGGAAGTAGTGTTCTTTTTTAGACCAAAGGCATTCACCCAGCTTTATTA
