# Supplementary material for: Identification of Genetic Loci for Sugarcane Leaf Angle at Different Developmental Stages by Genome-Wide Association Study
Source: Front Plant Sci. 2022 May 27;13:841693. doi: 10.3389/fpls.2022.841693 (PMC9185841; doi:10.3389/fpls.2022.841693)
Supplement: Supplementary Table 2 — Primer sets used in the PCR amplification. [file Table_2.DOCX]

**Supplemental Table S2 The primers used in the PCR amplification.**

| **Name** | **Sequence(5’-3’)** |
| --- | --- |
| 6A-1-F1 | TTACCAAATTTCACGTTTAACATTTCA |
| 6A-1-R1 | GTGCGGATAGCTGGCGTCC |
| 1A-1-F1 | TGTCTGCGGTGGGTGTGG |
| 1A-1-R1 | AAAAAGGGGGGAATCAAAGC |
| 4A-1-F1 | GCCTTGCTAGGGCATAGGC |
| 4A-1-R1 | AGCACTGCGAGGAGGAGATG |
| 5D-1-F1 | GGACATTACCAGAAGCGCTCTATC |
| 5D-1-R1 | TGGCTGCCACGGTGGCGG |
| 6A-2-F1 | GCGGATCTCGCTCCGTGG |
| 6A-2-R1 | TGCTGACTCAGAGCAACTCCAG |
| 7C-1-F1 | CTCAGCAGTACGCGGGCG |
| 7C-1-R1 | ACCACTGATCTCGTAGTAACTGGATT |
| 7D-1-F1 | CAAAAAGCTGTTCCTAATTTTTATTCTG |
| 7D-1-R1 | ATGAGCATCCCCGCCATT |
| 1A-2-F1 | TCTTTATTGGGTAATACCCATCGG |
| 1A-2-R1 | CACTCCCCTAGGAAGGCTGAC |
| 1A-3-F1 | CCGCCGTCGATGCCCTCC |
| 1A-3-R1 | GCCAGCCACCGGTAGGCG |
| 5A-1-F1 | GTAGCGCTAGGACAGTGGTTCTTT |
| 5A-1-R1 | ACATGATGTATCATACCCCCGAA |
| 5C-1-F1 | CCCACCGAGGACGTCTCG |
| 5C-1-R1 | ACTCAGCCCTAAGCCAGGAGA |
| 6C-1-F1 | TTTTTATCTGTCATCTGTGATCCTAGAA |
| 6C-1-R1 | TAATAAAGCTGGGTGAATGCCTT |
